# ID 342 - Perspectivas para o Tratamento Medicamentoso da Mpox

**DOI:** 10.5327/2237-9622.2025.v34s1.342

**Published:** 2025-11-25

**Authors:** Aramís Tupiná Alcântara de Moreira, Aline do Nascimento, Laís Nessa Neiva Pantuzza, Thaís Conceição Borges, Ana Carolina de Freitas Lopes, Luciene Fontes Schluckebier Bonan

**Keywords:** mpox, tecovirimat, MPXV, tratamento

## Abstract

**Introdução:**

A mpox é uma zoonose viral emergente, mas que atualmente é principalmente transmitida por contato humano-humano. A transmissão da infecção de humano-humano ocorre por contato com fluidos corporais e lesões na pele mucosas (boca, genitálias, secreções respiratórias e objetos contaminados). Os sintomas podem surgir entre 5 e 21 dias após o contato: febre, dor de cabeça, fadiga, dores musculares e nas costas; seguidos por erupção cutânea, linfadenopatia e formação de crostas. Pessoas com imunossupressão podem desenvolver complicações e morte por mpox. O diagnóstico laboratorial é possível por PCR (reação em cadeia da polimerase) de fluidos contidos nas lesões, esfregaços orofaríngeos, anais ou retais. Embora a varíola tenha sido erradicada em 1980, foi identificada a disseminação do MPXV (vírus causador da mpox) em humanos em maio de 2022 e novamente em 2024, quando a Organização Mundial da Saúde (OMS) voltou a declarar a mpox como Emergência de Saúde Pública de Importância Internacional (Espii), e então foram prorrogadas, até agosto de 2025, as recomendações sanitárias da OMS sobre a doença. Diante da emergência mundial, realizar a prospecção de tecnologias novas ou emergentes para o tratamento da doença e divulgar tais informações é considerada uma ação de relevância internacional. Assim, o objetivo deste trabalho é buscar tecnologias no horizonte para o tratamento da infecção por MPXV em todo o mundo, identificá-las e divulgar os resultados do seu desenvolvimento.

**Método:**

Foram realizadas buscas no ClinicalTrials.gov, em 5 de setembro de 2024, utilizando os termos: Recruiting, Not yet recruiting, Active, not recruiting, Completed, Enrolling by invitation Studies | Interventional Studies | Mpox OR, HereditaryMpox OR MPXV OR monkeypox | Phase 2, 3, 4, em desenvolvimento desde 2019.

**Resultados:**

Foram identificados cinco ensaios clínicos registrados no ClinicalTrials, que incluíram crianças recém-nascidas e pessoas imunossuprimidas: NCT05559099 (PALM007); NCT05597735 (UNITY); NCT05534165 (PLATINUM-CAN); NCT05534984 (STOMP); NCT06156566 (EPOXI). Foi identificada uma tecnologia em desenvolvimento: tecovirimat.

**Conclusão:**

Entre os cinco ensaios identificados, todos estão em andamento, são de fase 3 e 4, e apresentam previsão de conclusão a partir de setembro de 2024. Nota de imprensa recente do (NIH), financiador do ensaio PALM007, divulgou que o tecovirimat foi seguro, mas não melhorou as lesões decorrentes de mpox. Tecovirimat foi a única tecnologia avaliada na supramencionada busca do ClinicalTrials. Trata-se de um antiviral inibidor da VP37. O medicamento está disponível para uso desde 2022, porém sua efetividade havia sido avaliada apenas em estudos pré-clínicos, devido à raridade ou a aspectos éticos acerca da doença. No Brasil, a dispensa de registro foi aprovada pela Agência Nacional de Vigilância Sanitária (Anvisa) em 2022 e renovada em 2023 para aquisição exclusiva pelo Ministério da Saúde. Na Europa, a comercialização foi aprovada por autorização excepcional, condicionada a gerenciamento de risco anual. Enquanto nos Estados Unidos o acesso tem se dado por meio de acesso expandido do (CDC)ou ensaio clínico STOMP. Desse modo, ao passo que a sociedade científica produz, reúne e divulga dados sobre a efetividade e a segurança do tecovirimat em pessoas com mpox, o produto tem sido o único antiviral utilizado na expectativa de controle da doença no mundo.

